# Determining the Optimal SARS-CoV-2 mRNA Vaccine Dosing Interval for Maximum Immunogenicity

**DOI:** 10.7759/cureus.34465

**Published:** 2023-01-31

**Authors:** Michael Asamoah-Boaheng, David Goldfarb, Martin A Prusinkiewicz, Liam Golding, Mohammad E Karim, Vilte Barakauskas, Nechelle Wall, Agatha N Jassem, Ana Citlali Marquez, Chris MacDonald, Sheila F O’Brien, Pascal Lavoie, Brian Grunau

**Affiliations:** 1 Emergency Medicine, University of British Columbia, Vancouver, CAN; 2 Pathology and Laboratory Medicine, University of British Columbia, Vancouver, CAN; 3 Pediatrics, University of British Columbia, Vancouver, CAN; 4 Obstetrics and Gynecology, University of British Columbia, Vancouver, CAN; 5 Centre for Health Evaluation & Outcome Sciences, University of British Columbia, Vancouver, CAN; 6 Emergency Health Services, British Columbia Emergency Health Services, Vancouver, CAN; 7 Public Health Laboratory, British Columbia Centre for Disease Control, Vancouver, CAN; 8 Dalla Lana School of Public Health, University of Toronto, Toronto, CAN; 9 School of Epidemiology & Public Health, University of Ottawa & Canadian Blood Services, Ottawa, CAN; 10 Department of Pediatrics, University of British Columbia, Vancouver, CAN; 11 Emergency Medicine, St. Paul’s Hospital, University of British Columbia, Vancouver, CAN

**Keywords:** covid-19, covid-19 mrna vaccine, immunogenicity, spike total antibody concentrations, vaccine dosing interval, sars-cov-2

## Abstract

Objective: Emerging evidence indicates that longer SARS-CoV-2 vaccine dosing intervals results in an enhanced immune response. However, the optimal vaccine dosing interval for achieving maximum immunogenicity is unclear.

Methods: This study included samples from adult paramedics in Canada who received two doses of either BNT162b2 or mRNA-1273 vaccines and provided blood samples six months (170 to 190 days) after the first vaccine dose. The main exposure variable was vaccine dosing interval (days), categorized as “short” (first quartile), “moderate” (second quartile), “long” (third quartile), and “longest*” *interval (fourth quartile). The primary outcome was total spike antibody concentrations, measured using the Elecsys SARS-CoV-2 total antibody assay. Secondary outcomes included spike and receptor-binding domain (RBD) immunoglobulin G (IgG) antibody concentrations, and inhibition of angiotensin-converting enzyme 2 (ACE-2) binding to wild-type spike protein and several different Delta variant spike proteins. We fit a multiple log-linear regression model to investigate the association between vaccine dosing intervals and the antibody concentrations.

Results: A total of 564 adult paramedics (mean age 40 years, SD=10) were included. Compared to "short interval" (≤30 days), vaccine dosing intervals of the long (39-73 days) group (β= 0.31, 95% Confidence interval (CI): 0.10-0.52) and the longest (≥74 days) group (β = 0.82. 95% CI: 0.36-1.28) were associated with increased spike total antibody concentration. Compared to the short interval, the longest interval quartile was associated with higher spike IgG antibodies, while the long and longest intervals were associated with higher RBD IgG antibody concentrations. Similarly, the longest dosing intervals increased inhibition of ACE-2 binding to viral spike protein.

Conclusion: Increased mRNA vaccine dosing intervals longer than 38 days result in higher levels of anti-spike antibodies and ACE-2 inhibition when assessed six months after the first COVID-19 vaccine.

## Introduction

COVID-19, caused by the SARS-CoV-2 virus, was declared as a global pandemic on March 11, 2020, by the World Health Organization. SARS-CoV-2 vaccines were subsequently developed, with high efficacy for preventing short-term disease [1,2]. The two mRNA vaccines, BNT162b2 (Pfizer) and mRNA-1273 (Moderna), were tested and have been approved for use as a two-dose schedule, administered 21 and 28 days apart, respectively.

Existing evidence indicates that extended vaccine dosing intervals for mRNA [3-5] and Oxford-AstraZeneca [6] vaccines enhance vaccine immunogenicity. However, while previous studies have investigated specific intervals, the exact optimal dosing interval for achieving maximum immunogenicity is unclear. Further, while extended dosing intervals may lead to an enhanced post-vaccine immune response, the trade-off is sub-total immunity in the between-dose period. Thus, the shortest interval to achieve the highest long-term immune response would be the optimal scenario.

For these reasons, we sought to investigate a range of dosing intervals and resultant immunogenicity (measured at six months post first vaccine), focusing on the wild-type and delta variant strains, as delta variants were the predominant strains at the time of blood sampling. We hypothesized that the benefits in increasing immunogenicity with increasing dosing intervals would plateau at a certain dosing interval, and thus sought to identify the shortest interval to achieve the maximum post-vaccine immune response.

This article was previously posted to the bioRxiv preprint server for biology on March 4, 2022.

## Materials and methods

Study design and data source

We used samples from the COVID-19 Occupational Risks, Seroprevalence, and Immunity among Paramedics in Canada (CORSIP) observational cohort study. The CORSIP is a longitudinal prospective study examining the workplace risks and seroprevalence of SARS-CoV-2 exposure among paramedics (aged ≥ 19 years) working in the Canadian provinces of British Columbia, Ontario, Saskatchewan, and Manitoba. Study participants provided responses to an administered questionnaire including past medical history (i.e., hypertension, diabetes, asthma, chronic lung disease, chronic heart disease, liver disease, malignancy, and immunosuppression), data pertaining to results, and dates of vaccination and SARS-CoV-2 nucleic acid amplification tests (NAAT), and blood samples for serology testing. All samples used in this study were collected 170-190 days after the participant’s first vaccine dose. The CORSIP study was approved by the University of British Columbia and University of Toronto research ethics boards (IRB name: Providence Health Care Research Ethics Board; IRB approval number: H20-03620). The study obtained written informed consent from all subjects/participants before being enrolled in the study. 

Study participants

For this investigation, we included samples from CORSIP participants who: (1) had two doses of either BNT162b2 or mRNA-1273 vaccines; and (2) provided a blood sample six months ± 10 days (170-190 days) from the first vaccine date between December 16, 2020, and September 13, 2021. We excluded individuals who had previously tested positive for COVID-19 (defined as a positive NAAT COVID-19 test before the blood collection date or presence of anti-nucleocapsid antibodies in the blood (Roche, IND, USA)) given the known differential impact on antibody responses post-vaccination [7,8]. We also excluded cases for whom the second vaccine was > 130 days after the first, in order to separate the vaccine and the blood collection date by > 40 days, given the expected post-vaccine antibody surge [6,9].

Serological testing

All samples were tested with: (1) the Roche Nucleocapsid Elecsys Anti-SARS-Cov-2 (Roche, IND, USA) assay (to confirm eligibility); (2) the quantitative Roche Spike Elecsys Anti-SARS-Cov-2 S assay (Roche, IND, USA), which measures spike total antibody concentrations; (3) the V-PLEX COVID-19 Coronavirus Panel 2 immunoglobulin G (IgG) assay (Meso Scale Discovery, MD, USA), which measures IgG to the SARS-CoV-2 spike and receptor-binding domain (RBD) antigens; and (4) the V-PLEX SARS-CoV-2 Panel 19 ACE2 Kit (Meso Scale Discovery, MD, USA), which measures inhibition of angiotensin-converting enzyme 2 (ACE-2) binding to multiple delta (B.1.617.2) variant spike proteins.

Outcome measures

The primary outcome was spike total antibody concentrations, measured with the Elecsys assay. Secondary outcomes included spike and RBD IgG antibody concentrations (measured with the V-PLEX assay), and inhibition of ACE-2 binding to wild-type spike protein and several different Delta (B.1.617.2) variant spike proteins (AY.1, AY.2, B.1.617.2/AY.3/AY.5/AY.6/AY.7/AY.14, B.1.617.2/AY.4, and AY.12).

Statistical analysis

We performed statistical analyses with SPSS (IBM, USA) and R (V. 3.6.1) software. For participant characteristics, we described categorical variables as counts (with percentages) and continuous variables as mean (with SD) or median (with interquartile range (IQR)) values. To visualize the trend of the antibody response over the range of dosing intervals, we created: (1) created scatter-plots with cubic spline curves (with 95% confidence intervals); and (2) error plots of the means (with 95% confidence intervals) of outcomes values stratified by incremental one-week vaccine dosing intervals, as well as within subgroups defined by vaccine type [10].

To investigate the relationship between vaccine dosing intervals and antibody outcomes, we divided participants into vaccine dosing interval quartiles: short interval (first quartile), moderate interval (second quartile), long interval (third quartile), and longest interval (fourth quartile). We used the four categories of the vaccine dosing interval in our multivariate regression analysis instead of the nine groups used for the error plots to avoid overfitting of the model as too many dummy variables would have been introduced in the model. We compared characteristics and outcomes between quartiles using Analysis of Variance (ANOVA) to test for differences in the mean ages of participants, Chi-square test to test for differences between categorical variables, and the Kruskal Wallis H test for the difference between outcome measures.

We fit a multiple log-linear regression model to estimate the association between the dosing interval quartiles (with the first quartile as the reference) and both the primary and secondary outcome variables, adjusted for type of vaccine, interval between the second vaccine and blood collection date (days), sex, age, and medical history (each past medical history category was modeled as a binary variable). As sensitivity analyses, we repeated the primary analyses incorporating an interaction term including dosing interval category and vaccine type, in order to determine if vaccine type modifies the association of vaccine dosing intervals and immunogenicity. Similarly, we repeated the primary analysis after incorporating an interaction between “dosing interval category” and “interval between second vaccine and blood collection draw date” to determine if model results were modified based on the time proximity of the blood collection to the second vaccine.

## Results

The study included a total of 564 adult paramedics with a mean age of 40 years. 253 (45%) were female sex at birth and 79 (14%) reported a race from Asia and other ethnic groups (Table 1). Overall, 469 (83%) received two doses of the BNT162b2 vaccine and 95 (17%) received two doses of the mRNA-1273 SARS-CoV-2 vaccine.

**Table 1:** Study characteristics and serology results of the six-month sample Chi-square test was used to test for the significant difference between the categorical variables and the dosing interval, ANOVA for the differences in the mean ages of participants, Kruskal-Wallis H test for the difference between the various antibodies. IQR: Interquartile range; RBD: Receptor-binding domain; IgG: Immunoglobulin G; ACE-2: Angiotensin-converting enzyme 2; gM: Geometric mean; gSD: Geometric standard deviation; n, number; Ab: antibody; 2nd vac-to-BC: Interval from Vaccine 2 to blood collection date; ANOVA: Analysis of variance

Study variables	Full Cohort, n = 564	Dosing Interval (days)				P-value
		Short (≤30 days)	Moderate (31-38 days)	Long (39-73 days)	Longest (≥ 74days)	
		n=135	n = 156	n = 134	n =139	
Age (years), mean (SD)	40 (10)	38 (9.0)	39 (10)	41 (9.0)	42 (12)	0.006
Female Sex, n (%)	253 (45)	63 (47)	73 (47)	52 (39)	65 (47)	
Male Sex, n (%)	293 (52)	71 (53)	83 (53)	78 (58)	61 (44)	0.316
Prefer not to answer, n (%)	18 (3.0)	1.0 (1.0)	0.0	4.0 (3.0)	13 (9.0)	
Ethnicity/Race, n (%)						
White	485 (86)	111 (82)	133 (85)	120 (90)	121 (87)	0.075
Asian	36 (6.0)	11 (8.0)	15 (10)	5.0 (4.0)	5.0 (4.0)	
Other	43 (8.0)	11 (8.0)	8.0 (5.0)	7.0 (5.0)	17 (12)	
Educational level, n (%)						
Non-university, n (%)	323 (57)	73 (54)	83 (53)	88 (66)	79 (57)	0.020
University Bachelor’s degree, n (%)	202 (36)	58 (43)	70 (45)	35 (26)	39 (28)	
University Graduate degree, n (%)	18 (3.0)	3.0 (2.0)	3.0 (2.0)	5.0 (4.0)	7.0 (5.0)	
Others, n (%)	3.0 (1.0)	0.0	0.0	2.0 (2.0)	1.0 (1.0)	
Smoking History, n (%)						
Cigarette use	32 (6.0)	8.0 (6.0)	9.0 (6.0)	6.0 (5.0)	9.0 (7.0)	0.690
E-cigarette	11 (2.0)	4.0 (3.0)	3.0 (2.0)	1.0 (1.0)	3.0 (2.0)	0.636
Medical history, n (%)						
Hypertension	45 (8.0)	7.0 (5.0)	17 (11)	13 (10)	8.0 (6.0)	0.203
Diabetes	10 (2.0)	2.0 (2.0)	2.0 (1.0)	3.0 (2.0)	3.0 (2.0)	0.901
Asthma	75 (13)	15 (12)	28 (18)	15 (12)	17 (13)	0.296
Chronic lung disease	4.0 (1.0)	2.0 (2.0)	0.0	2.0 (2.0)	0.0	0.218
Chronic heart disease	4.0 (1.0)	1.0 (1.0)	2.0 (1.0)	1.0 (1.0)	0.0	0.637
Liver disease	8.0 (2.0)	1.0 (1.0)	3.0 (2.0)	3.0 (2.0)	1.0 (1.0)	0.609
Malignancy	13 (2.0)	2.0 (2.0)	3.0 (2.0)	3.0 (2.0)	5.0 (4.0)	0.667
Immune suppressed	1.0 (1.0)	3.0 (2.0)				
Vaccine Type, n (%)						
BNT162b2	469 (83)	124 (92)	151 (97)	73 (54)	121 (87)	0.000
mRNA-1273	95 (17)	11 (8.0)	5.0 (3.0)	61 (46)	18 (13)	
2nd vac-to-BC, median (IQR), days	143 (109,151)					
Serological testing, gm (gSD)						
Total spike Ab (U/mL)	1970 (3.0)	1058 (2.0)	1240 (2.0)	1845(3.0)	6448 (2.0)	0.000
Spike IgG (Au/mL)	44311(2.0)	29016 (2.0)	30669 (2.0)	45950 (2.0)	97554 (2.0)	0.000
RBD IgG (Au/mL)	24818 (3.0)	14839 (2.0)	16739 (2.0)	24683(3.0)	63971 (2.0)	0.000
ACE2 inhibition						
Spike (Wuhan)	9.0 (3.0)	5.0 (2.0)	6.0 (2.0)	9.0 (3.0)	27 (3.0)	0.000
Spike (AY.1)	9.0 (3.0)	5.0 (2.0)	6.0 (2.0)	9.0 (2.0)	26 (3.0)	0.000
Spike (AY.12)	9.0 (3.0)	5.0 (2.0)	6.0 (2.0)	8.0 (2.0)	23 (3.0)	0.000
Spike (AY.2)	9.0 (3.0)	5.0 (2.0)	6.0 (2.0)	8.0 (2.0)	24 (3.0)	0.000
Spike (B.1.617.2; AY.3; AY.5; AY.6; AY.7; AY.14)	9.0 (3.0)	6.0 (2.0)	6.0 (2.0)	9.0 (2.0)	26 (3.0)	0.000
Spike (B.1.617.2; AY.4)	10 (3.0)	6.0 (2.0)	6.0 (2.0)	9.0 (2.0)	26 (3.0)	0.000

The median vaccine dosing interval was 38 days (IQR 31-73), and thus cases were classified as short interval (≤30 days), moderate interval (31-38 days), long interval (39-73 days), and longest interval (≥74 days) based on the estimated quartiles. The quartiles vaccine dosing interval were estimated to ensure adequate sample were represented in each category. Table 1 shows participant characteristics, which were similar across dosing interval quartiles. All outcome measures increased with each vaccine dosing interval quartile.

**Figure 1:**
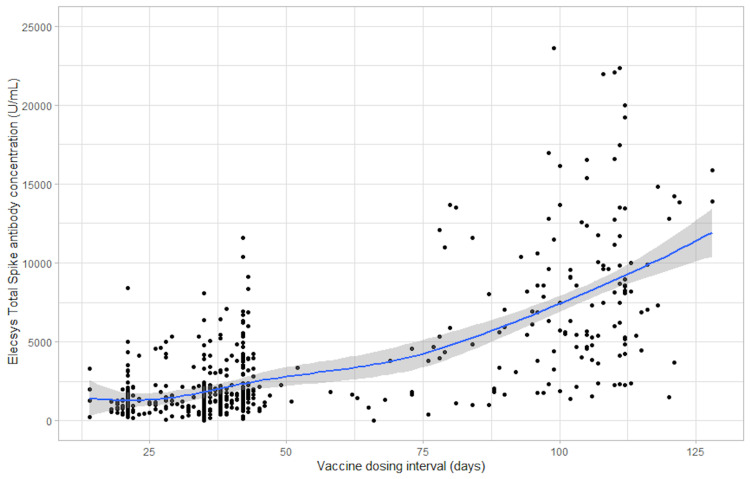
Scatter plot of the Elecsys spike total antibody concentration (U/mL) against the vaccine dosing interval (days)

**Figure 2:**
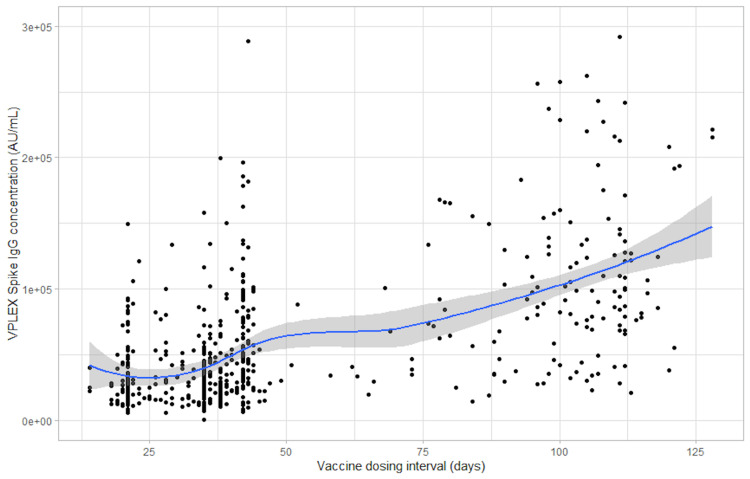
Scatter plot of the V-PLEX spike IgG antibody concentration (AU/mL) against the vaccine dosing interval (days) IgG: Immunoglobulin G

**Figure 3:**
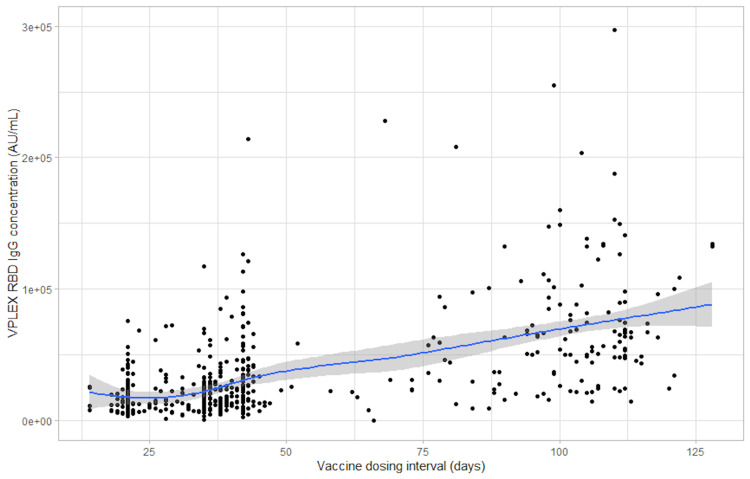
Scatter plot of the V-PLEX RBD antibody concentration (AU/mL) against the vaccine dosing interval (days) RBD: Receptor-binding domain

Figures 1, 2, and 3 show scatter plots of the anti-spike total antibody concentration (U/mL), the anti-spike IgG antibody concentration, and the anti-RBD antibody concentration, respectively, each against the vaccine dosing interval (days). Supplementary figures 4, 6, 8, show mean error plots to describe the relationship between the outcome measures over a range of vaccine dosing intervals, demonstrating an increase in all measures of immunogenicity with increasing dosing intervals. A similar relationship was seen among individuals who were fully vaccinated with either BNT162b2 and mRNA-1273 vaccines (see Supplementary Figures 5 and 7).

The primary regression analyses demonstrated that with reference to the first quartile (low interval), the long and longest dosing intervals were all associated with increased log total spike antibody concentrations (Table 2, Table 4). Models examining secondary outcomes of anti-spike RBD IgG showed that the long and the longest quartiles, with reference to the first quartile, were associated with significantly increased log antibody concentrations. Models examining secondary outcomes of ACE-2 inhibition to the wild type spike protein and Delta strain spike variant proteins showed similar results to the main analysis (see Table 3).

**Table 2:** Multivariate analysis (multiple log-linear regression) of the association between vaccine dosing intervals and antibody immune response Model 1: Outcome variable is Elecsys spike total antibody concentration; Model 2: Outcome variable is V-PLEX spike IgG antibody concentration; Model 3: Outcome variable is V-PLEX RBD antibody concentration. Low dosing interval (< 31 days) was set as a reference category. ** indicates model is significant at p < 0.05 β: Regression coefficient; CI: Confidence interval; RBD: Receptor-binding domain; IgG: Immunoglobulin G All models were adjusted for type of vaccine administered, age, sex at birth, time from second vaccine to blood collection date, and comorbidities (hypertension, diabetes, asthma, chronic lung diseases, heart diseases, kidney diseases, liver diseases, cancer, and immune suppressed).

Dosing intervals	Model 1		Model 2		Model 3	
	Coeff ( β )	95% CI	Coeff (β)	95% CI	Coeff (β)	95% CI
Moderate (31-38 days)	0.17	(-0.0060, 0.35)	-0.01	(-0.19, 0.17)	0.11	(-0.092, 0.30)
Long (39-73 days)	0.31	(0.10, 0.52) **	0.16	(-0.05, 0.38)	0.25	(0.017, 0.49) **
Longest (≥74days)	0.82	(0.36, 1.28) **	0.77	(0.31, 1.23) **	1.20	(0.69, 1.71) **

**Table 3:** Multivariate analysis (multiple log-linear regression) of the association between vaccine dosing intervals and secondary antibody outcomes Model 1: Outcome variable is “Wild-type”; Model 2: Outcome variable is “Spike (AY.1)”; Model 3: Outcome variable is “Spike (AY.12)”; Model 4: Outcome variable is “Spike (AY.2)”; Model 5: Outcome variable is Spike (B.1.617.2; AY.3; AY.5; AY.6; AY.7; AY.14); Model 6: Outcome variable is Spike (B.1.617.2; AY.4). Low dosing interval (< 31 days) was set as a reference category. ** indicates model is significant at p < 0.05 β: Regression coefficient; CI: Confidence interval All models were adjusted for type of vaccine administered, age, “sex at birth”, time from second vaccine to blood collection date, and comorbidities (hypertension, diabetes, asthma, chronic lung diseases, heart diseases, kidney diseases, liver diseases, cancer, and immune suppressed).

Dosing intervals	Model 1	Model 2	Model 3	Model 4	Model 5	Model 6
	β (95% CI)	β (95% CI)	β (95% CI)	β (95% CI)	β (95% CI)	β (95% CI)
Moderate (31-38 days)	0.090(-0.15, 0.33)	0.12 (-0.087, 0.0.32)	0.071 (-0.15, 0.29)	0.11 (-0.089, 0.32)	0.092 (-0.13, 0.31)	0.069 (-0.15, 0.29)
Long (39-73 days)	0.27 (-0.023, 0.55)	0.25 (0.0040, 0.50) **	0.23 (-0.028, 0.50)	0.23 (-0.011, 0.47)	0.23 (-0.031, 0.50)	0.23 (-0.025, 0.49)
Longest (≥74days)	1.24 (0.61, 1.87) **	1.17 (0.64, 1.71) **	1.080 (0.51, 1.65) **	1.13 (0.80, 1.65) **	1.06 (0.49, 1.64) **	1.08 (0.53, 1.64) **

In the two sensitivity analyses, we separately introduced interaction terms “vaccine type & vaccine dosing interval” and “vaccine dosing interval & interval between second dose and blood collection date” into regression models. However, none were significant (Tables 5, 6, and 7). 

## Discussion

We investigated immunogenicity at six months after the first COVID-19 vaccine among 564 adults who received SARS-COV-2 mRNA vaccines to identify the vaccine dosing interval for achieving maximum immunogenicity. We found that longer vaccine dosing intervals (> 38 days) resulted in higher measures of immunogenicity six months post vaccination. While we had hypothesized that the rise in immune measures would plateau at a particular point, antibody levels continued to rise with extended vaccine intervals up to 130 days. This data will assist clinicians and policy-makers by demonstrating that increasing vaccine dosing intervals (up to 130 days) results in corresponding increased six-month immunogenicity.

Our data demonstrated a continuous increasing trend of immunogenicity as the vaccine dosing interval increased, which was consistent with all immune measures. Our regression models were consistent. In deciding the optimal vaccine dosing interval, there is a trade-off between decreased immunity between the first and second dose with longer dosing intervals and enhanced immunity in the post-second dose period. We had hoped to identify a plateau in the immunogenicity measures with increasing dosing intervals. However, this was not apparent. Instead, public health providers need to balance these two competing priorities, the balance of which may change based on the incidence of COVID-19 in the community, and the risk of severe disease in individual patients. Of additional consideration is the availability of booster (third) vaccine dosing, i.e., if readily available then decreased immunity in the post-second dose period among those with short vaccine dosing intervals can be addressed by a booster dose [11,12]. 

Community level-immunity may also play a role in vaccine dosing interval decisions. Previous modeling studies have demonstrated that extending SARS-CoV-2 vaccine dosing intervals results in reduced cumulative mortality. This is due to earlier access to the first vaccine dose, even without considering the long-term benefit of post-second dose enhanced immunogenicity [13,14]. Global-level immunity and vaccine distribution also deserve consideration. While booster doses may serve as a method to augment waning immunity in the months after the second mRNA dose, diverting vaccine supplies to low- and middle-income countries where only 10.4% of the population has received at least one dose of the vaccine would be key to improve vaccination coverage. [15]. Lengthening between-dose intervals may result in more robust longer-term immunity, thus allowing for delayed booster doses, and re-direction of vaccine supplies to under-served regions.

Our results are consistent with recently released (pre-peer review) data, showing that a seven-eight-week interval between doses improved vaccine effectiveness in comparison to a three-four-week interval [16]. Our data also corroborates immunogenicity studies that compared an extended dosing interval schedule of the BNT162b2 vaccine to the standard schedule (three weeks) [4,5]. A recent study by Parry et al. found that delaying or extending the timing of the second dose of the BNT162b2 vaccine up to 12 weeks generated higher antibody levels with 3.5-fold higher peak antibody values than the standard three-week interval [17]. Additionally, higher two dose vaccine effectiveness was observed when a dosing interval of more than six weeks was adopted between BNT162b2 doses compared to the standard schedule [18]. Similar findings were found in studies conducted by Moghadas et al and Payne et al [3,15].

Some jurisdictions such as the United States administer the second dose of the SAR-COV-2 vaccines by the stipulated three- or four-week schedules for Pfizer-BioNTech and Moderna vaccines, respectively. Few countries, including the UK and parts of Canada have approved guidelines for delaying the second dose schedules of the vaccines to up to 12 and 16 weeks respectively [19,20]. The existence of these varying dosing interval strategies raises some concerns as to which dosing interval is optimal in achieving maximal immunogenicity. Our study sought to investigate this phenomenon to identify the minimum optimal threshold to improve immunity against COVID-19. We found that increasing dosing intervals resulted in a continual increase in immunogenicity measures at six months.

Limitations

This was an observational study, and it is possible that unaccounted for confounders affected our results. We measured antibody concentrations at six months from the start of the vaccine series, which provides health policy leaders a comparison of different vaccination strategies, showing differences in immunogenicity at six months. Previous data has shown that antibody levels initially increase and then decrease over time. By virtue of a fixed time juncture of outcome ascertainment, the longer vaccine dosing interval groups had the shortest interval from the second vaccine to blood collection. Although we adjusted for the second vaccine-to-blood collection interval in our mode, residual confounding may have played a role in our results. We excluded cases with previous COVID-19 using nucleocapsid serological testing-which, although a high-performance test 7, may have mis-classified samples. However, we also used a polymerase chain reaction (PCR) test to identify previous COVID-19 and to validate cases already identified with the nucleocapsid serological testing to minimize a potential sample misclassification. Our study participants consist of paramedics that were primarily healthy, middle aged, and white, and thus these data may not be generalizable to other groups. Although antibody levels have been correlated with vaccine effectiveness, we did not examine clinical endpoints such as break-through infections or severe disease [21].

## Conclusions

Compared to short intervals (≤ 30 days), the long (39-73 days) and longest ( (≥ 74 days) vaccine dosing intervals were associated with increased spike total antibody concentration. Thus, extending the interval between SARS-CoV-2 mRNA vaccine doses from 39 to 123 days is associated with a continuous increase in immunogenicity when measured at six months after the first COVID-19 vaccine dose. 

## Figures and Tables

**Figure 4:**
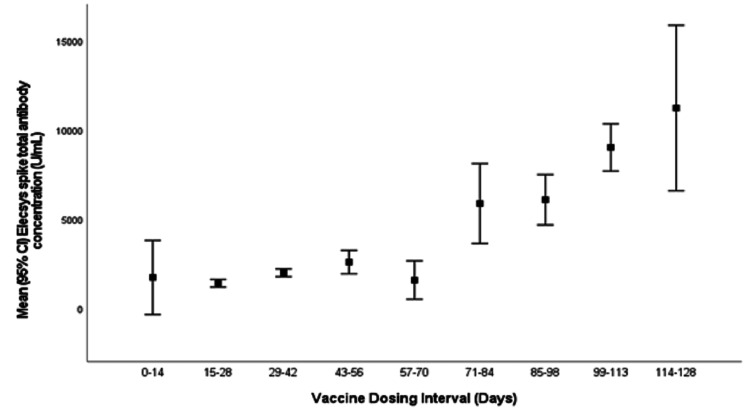
Error plots of Elecsys spike total antibody concentration versus the dosing interval (days)

**Figure 5:**
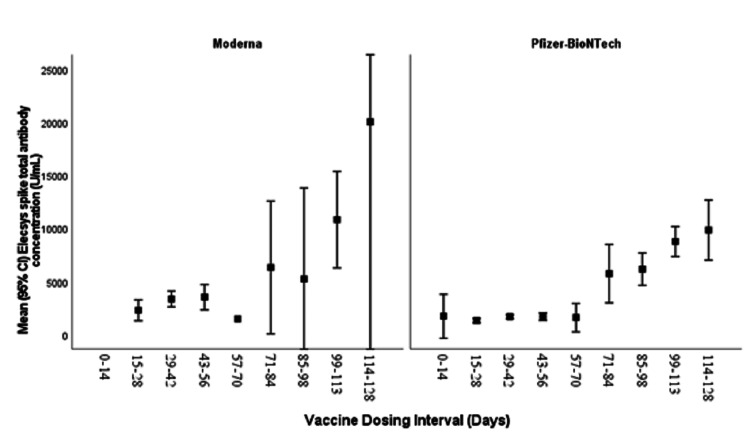
Error plots of Elecsys spike total antibody concentration versus the dosing interval (days), stratified by the vaccine type administered

**Figure 6:**
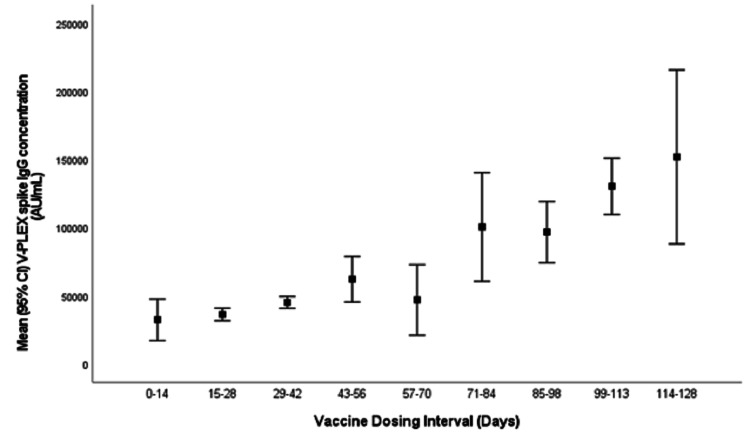
Error plots of V-PLEX spike IgG antibody concentration versus the vaccine dosing interval (days) IgG: Immunoglobulin G

**Figure 7:**
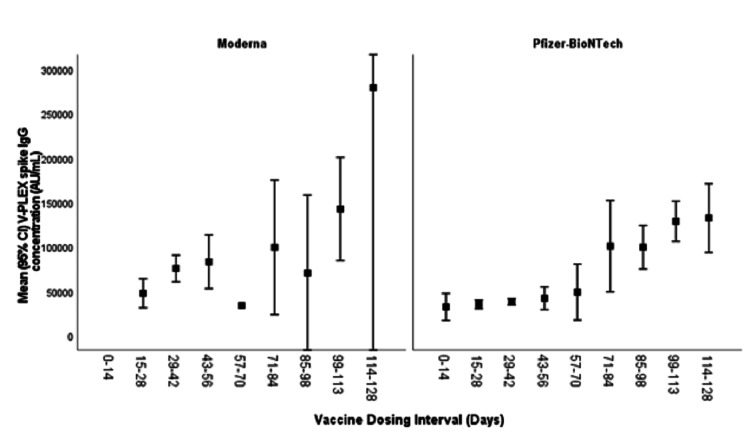
Error plots of V-PLEX spike IgG antibody concentration versus the dosing interval (days), stratified by the type of vaccine administered IgG: Immunoglobulin G

**Figure 8:**
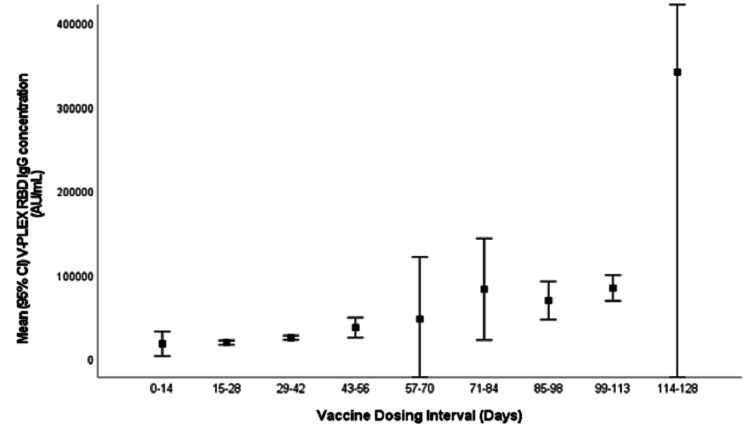
Error plots of V-PLEX RBD IgG concentration versus the dosing interval (days) RBD: Receptor-binding domain; IgG: Immunoglobulin G

**Figure 9:**
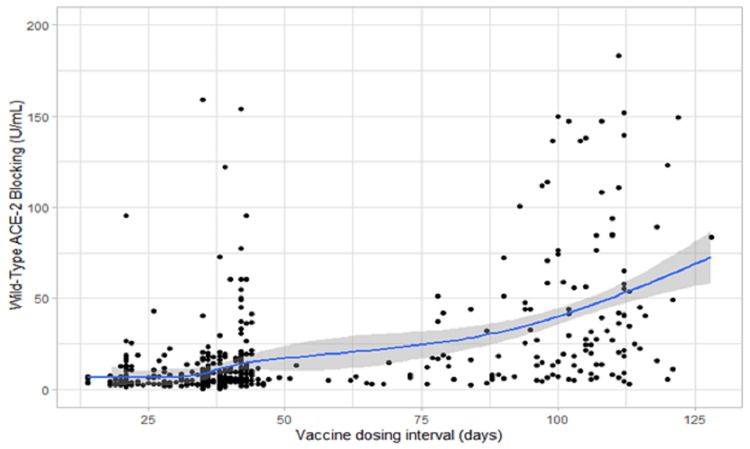
Scatter plot of the Wild-type ACE-2 inhibition concentration against the vaccine dosing interval (days) ACE-2: Angiotensin-converting enzyme 2

**Figure 10:**
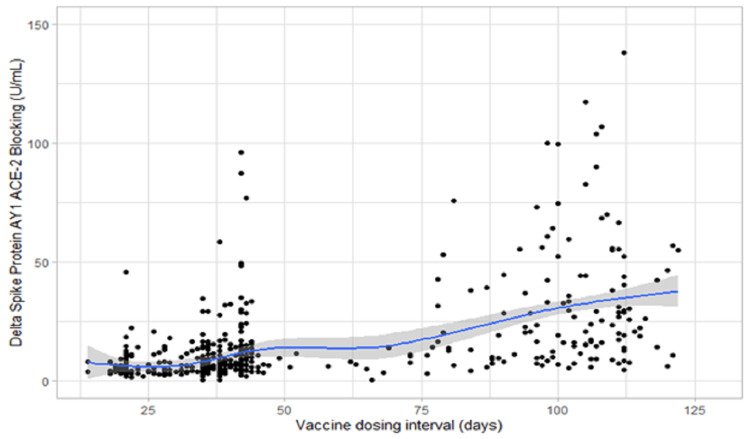
Scatter plot of the delta spike protein AY1 ACE-2 inhibition concentration against the vaccine dosing interval (days) ACE-2: Angiotensin-converting enzyme 2

**Figure 11:**
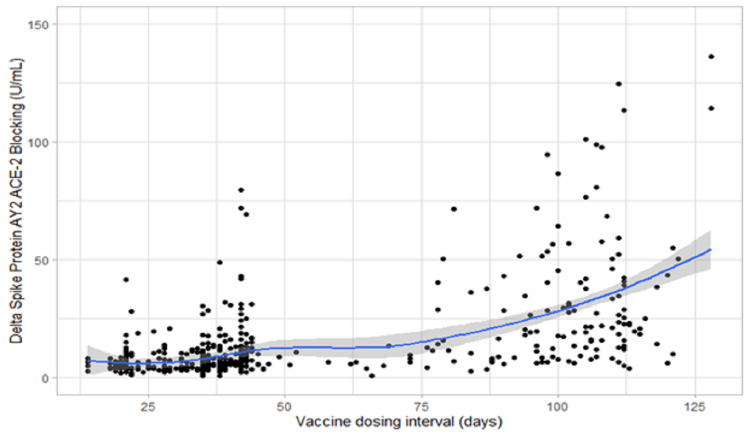
Scatter plot of the delta spike protein AY2 ACE-2 inhibition concentration against the vaccine dosing interval (days) ACE-2: Angiotensin-converting enzyme 2

## Appendices

**Table 4:** Multivariate analysis (multiple log-linear regression) of the association between vaccine dosing intervals and antibody immune response (full output) Model 1: Outcome variable is Elecsys spike total antibody concentration; Model 2: Outcome variable is V-PLEX spike IgG antibody concentration; Model 3: Outcome variable is V-PLEX RBD antibody concentration. β: Regression coefficient; CI: Confidence interval; 2nd vac-to-BC interval: Interval between second vaccine and blood collection date (in days); IgG: Immunoglobulin G; RBD: Receptor-binding domain **indicates model is significant at p < 0.05.

Variables	Model 1		Model 2		Model 3	
	Coeff (β)	95% CI	Coeff (β)	95% CI	Coeff (β)	95% CI
Main exposure (dosing Intervals)						
Moderate (31-38 days)	0.17	(-0.0060, 0.35)	-0.01	(-0.19, 0.17)	0.11	(-0.092, 0.30)
Long (39-73 days)	0.31	(0.10, 0.52) **	0.16	(-0.05, 0.38)	0.25	(0.017, 0.49) **
Longest (≥74days)	0.82	(0.36, 1.28) **	0.77	(0.31, 1.23) **	1.20	(0.69, 1.71) **
Adjusted variables						
Age (per year)	-0.010	(-0.016, -0.004) **	-0.013	(-0.019, -0.0070) **	-0.015	(-0.022, -0.0090) **
Sex at birth (male vs others)	0.070	(-0.046, 0.19)	0.10	(-0.012, 0.22)	0.090	(-0.040, 0.22)
Vaccine type (BNT162b2 vs mRNA-1273)	-0.37	(-0.54, -0.21) **	-0.51	(-0.68, 0.34) **	-0.55	(-0.74, -0.36) **
2nd vac-to-BC interval (days)	-0.0010	(-0.006, 0.004)	-0.0060	(-0.011, -0.001) **	-0.0040	(-0.010, 0.0020)
Past Medical history						
Hypertension	-0.16	(-0.40, 0.086)	-0.17	(-0.42, 0.070)	-0.14	(-0.41, 0.14)
Diabetes	-0.066	(-0.56, 0.43)	-0.19	(-0.68, 0.30)	-0.18	(-0.72, 0.38)
Asthma	0.032	(-0.14, 0.20)	0.046	(-0.12, 0.21)	-0.0070	(-0.19, 0.18)
Chronic lung disease	-0.37	(-1.09, 0.35)	-0.52	(-1.25, 0.20)	-0.57	(-1.38, 0.24)
Chronic heart disease	-0.35	(-1.11, 0.41)	-0.39	(-1.13, 0.39)	-0.38	(-1.23, 0.47)
Kidney disease	-0.15	(-1.39, 1.09)	-0.77	(-2.01, 0.48)	-0.67	(-2.06, 0.72)
Liver disease	-0.33	(-0.80, 0.15)	-0.45	(-0.92, 0.03)	-0.42	(-0.95, 0.12)
Malignancy	0.022	(-0.35, 0.39)	-0.18	(-0.55, 0.19)	-0.17	(-0.58, 0.24)
Immune suppressed	-1.30	(-1.71, -0.90)	-0.67	(-1.07, -0.27)	-1.28	(-1.73, -0.84)

**Table 5:** Multiple log-linear regression investigating association between dosing intervals, interaction term, and antibody response Model 1: Outcome variable is Elecsys spike total antibody concentration; Model 2: Outcome variable is V-PLEX spike IgG antibody concentration; Model 3: Outcome variable is V-PLEX RBD antibody concentration Low dosing interval (< 31 days) was set as a reference category. **indicates model is significant at p < 0.05. β: Regression coefficient; CI: Confidence interval; RBD: Receptor-binding domain; IgG: Immunoglobulin G All models were adjusted for type of vaccine administered, age, “sex at birth”, time from second vaccine to blood collection date, and comorbidities (hypertension, diabetes, asthma, chronic lung diseases, heart diseases, kidney diseases, liver diseases, cancer, and immune suppressed).

Covariates	Model 1			Model 2			Model 3		
	Coeff (β)	95% CI	p-value	Coeff (β)	95% CI	p-value	Coeff (β)	95% CI	p-value
Interaction term									
Moderate int*vaccine type	-0.028	(-0.73, 0.67)	0.94	-0.43	(-1.13, 0.27)	0.23	-0.38	(-1.17, 0.40)	0.34
Long int*vaccine type	0.097	(-0.38, 0.57)	0.69	-0.29	(-0.77, 0.18)	0.23	-0.35	(-0.88, 0.18)	0.20
Longest int*vaccine type	0.34	(-0.20, 0.88)	0.21	0.032	(-0.51, 0.57)	0.91	0.063	(-0.54, 0.67)	0.84

**Table 6:** Multiple log-linear regression investigating association between dosing intervals, interaction term, and ACE-2 inhibition Model 1: Outcome variable is Wild-type; Model 2: Outcome variable Spike (AY.1); Model 3: Outcome variable is Spike (AY.12); Model 4: Outcome variable is Spike (AY.2); Model 5: Outcome variable is Spike (B.1.617.2; AY.3; AY.5; AY.6; AY.7; AY.14); and Model 6: Outcome variable is Spike (B.1.617.2; AY.4). Low dosing interval (< 31 days) was set as a reference category * indicates model is significant at p < 0.05. β: Regression coefficient; CI: Confidence interval All models were adjusted for type of vaccine administered, age, “sex at birth”, time from second vaccine to blood collection date, and comorbidities (hypertension, diabetes, asthma, chronic lung diseases, heart diseases, kidney diseases, liver diseases, cancer, and immune suppressed).

	Model 1 β (95% CI)	Model 2 β (95% CI)	Model 3 β (95% CI)	Model 4 β (95% CI)	Model 5 β (95% CI)	Model 6 β (95% CI)
Interaction term						
Moderate int*vaccine type	-0.28 (-1.22, 0.67)	-0.40 (-1.21, 0.41)	-0.42 (-1.28, 0.44)	-0.31 (-1.11, 0.48)	-0.43 (-1.30, 0.44)	-0.37 (-1.22, 0.48)
Long int*vaccine type	-0.14 (-0.78, 0.50)	-0.24 (-0.79, 0.30)	-0.28 (-0.86, 0.30)	-0.21 (-0.75, 0.33)	-0.26 (-0.85, 0.33)	-0.23 (-0.81, 0.34)
Longest int*vaccine type	0.42 (-0.33, 1.17)	0.20 (-0.43, 0.84)	0.26 (-0.42, 0.93)	0.24 (-0.38, 0.86)	0.27 (-0.42, 0.95)	0.24 (-0.42, 0.91)

**Table 7:** Multiple log-linear regression investigating association between dosing intervals, interaction term between 2nd vac-to-BC interval, and antibody response Model 1: Outcome variable in is Wild-type; Model 2: Outcome variable is Spike (AY.1); Model 3: Outcome variable is Spike (AY.12); Model 4: Outcome variable is Spike (AY.2); Model 5: Outcome variable is Spike (B.1.617.2; AY.3; AY.5; AY.6; AY.7; AY.14); and Model 6: Outcome variable is Spike (B.1.617.2; AY.4). Low dosing interval (< 31 days) was set as a reference category. * indicates model is significant at p < 0.05. β: Regression coefficient; CI: Confidence interval; 2nd vac-to-BC: Interval between second vaccine and blood collection date (in days). All models were adjusted for type of vaccine administered, age, “sex at birth”, time from second vaccine to blood collection date, and comorbidities (hypertension, diabetes, asthma, chronic lung diseases, heart diseases, kidney diseases, liver diseases, cancer, and immune suppressed).

Covariates	Model 1			Model 2			Model 3		
	Coeff (β)	95% CI	p-value	Coeff (β)	95% CI	p-value	Coeff (β)	95% CI	p-value
Interaction term									
Moderate*2nd vac-to-BC	0.0010	(-0.015, 0.017)	0.895	-0.0050	(-0.021, 0.011)	0.531	-0.0060	(-0.024, 0.011)	0.472
Long *2nd vac-to-BC	0.016	(-0.0030, 0.036)	0.100	-0.006	(-0.026, 0.013)	0.525	0.0060	(-0.016, 0.027)	0.617
Longest * 2nd vac-to-BC	0.000079	(-0.016, 0.016)	0.992	-0.018	(-0.035, -0.0020)	0.025	-0.017	(-0.035, 0.001)	0.067
